# Mitochondrial Genome of the Stonefly *Kamimuria wangi* (Plecoptera: Perlidae) and Phylogenetic Position of Plecoptera Based on Mitogenomes

**DOI:** 10.1371/journal.pone.0086328

**Published:** 2014-01-23

**Authors:** Qian Yu-Han, Wu Hai-Yan, Ji Xiao-Yu, Yu Wei-Wei, Du Yu-Zhou

**Affiliations:** 1 School of Horticulture and Plant Protection and Institute of Applied Entomology, Yangzhou University, Yangzhou, Jiangsu, China; 2 College of Forestry, Southwest Forestry University, Kunming, Yunnan, China; The University of Hong Kong, China

## Abstract

This study determined the mitochondrial genome sequence of the stonefly, *Kamimuria wangi*. In order to investigate the relatedness of stonefly to other members of Neoptera, a phylogenetic analysis was undertaken based on 13 protein-coding genes of mitochondrial genomes in 13 representative insects. The mitochondrial genome of the stonefly is a circular molecule consisting of 16,179 nucleotides and contains the 37 genes typically found in other insects. A 10-bp poly-T stretch was observed in the A+T-rich region of the *K. wangi* mitochondrial genome. Downstream of the poly-T stretch, two regions were located with potential ability to form stem-loop structures; these were designated stem-loop 1 (positions 15848–15651) and stem-loop 2 (15965–15998). The arrangement of genes and nucleotide composition of the *K. wangi* mitogenome are similar to those in *Pteronarcys princeps*, suggesting a conserved genome evolution within the Plecoptera. Phylogenetic analysis using maximum likelihood and Bayesian inference of 13 protein-coding genes supported a novel relationship between the Plecoptera and Ephemeroptera. The results contradict the existence of a monophyletic Plectoptera and Plecoptera as sister taxa to Embiidina, and thus requires further analyses with additional mitogenome sampling at the base of the Neoptera.

## Introduction

The order Plecoptera is comprised of 16 families and includes 3,497 species [1]; these occur on all continents except Antarctica [2]. Plecoptera is a small order of hemimetabolous insects that are primarily associated with clean, cool, running water and cool, damp terrestrial environments [3]. The nymphs generally have their highest density in riffle areas of streams where rocks, gravel, snags, and accumulated leaves are abundant. Their potential use as biological indicators of water quality is well-known [4].

The insects mitochondrial genome is a circular molecule ranging from 15 to 20 kp; it generally encodes two rRNA genes, 22 tRNA genes, 13 protein-coding genes (PCGs), and an A+T-rich region varying in length among taxa [5], [6]. Recently, whole mitochondrial DNA (mtDNA) sequences have been used in phylogenetic analyses of various insect orders. After the complete mitochondrial genome of *Pteronarcys princeps* was published [7], increasing numbers of researchers used this data for phylogenetic analyses, which resulted in various theories on the phylogenetic position of Plecoptera. Zhang et al. and Lin et al. [8], [9] used 13 PCGs of mtDNA data to support relatedness between Ephemeroptera and Plecoptera within the Neoptera.

The stoneflies are an ancient group of insects. Their fossil record extends into the Lower Permian [10]. But the phylogenetic position of Plecoptera has long been under debate. Some of the competing evolutionary hypotheses place the stoneflies in a single basal group, diverging early after the split between the Paleoptera and Neopteran ancestors, and before other Neopteran diversification [11], [12]. At the same time, the relationship about Styloperlidae, Peltoperlidae, Chloroperlidae, Perlidae and Perlodidae was controversial [13], [14]. The aim of this study was to discuss the phylogenetic position of Plecoptera based on mtDNA.

In this work, we present the complete mitochondrial genome of the stonefly, *Kamimuria wangi* Du, 2012 (Perlidae) and reconstructed phylogenies of 17 major basal Pterygote lineages based on 13 PCGs obtained from mitochondrial genomes.

## Results and Discussion

### Genome organization, structure and composition

**Figure 1:**
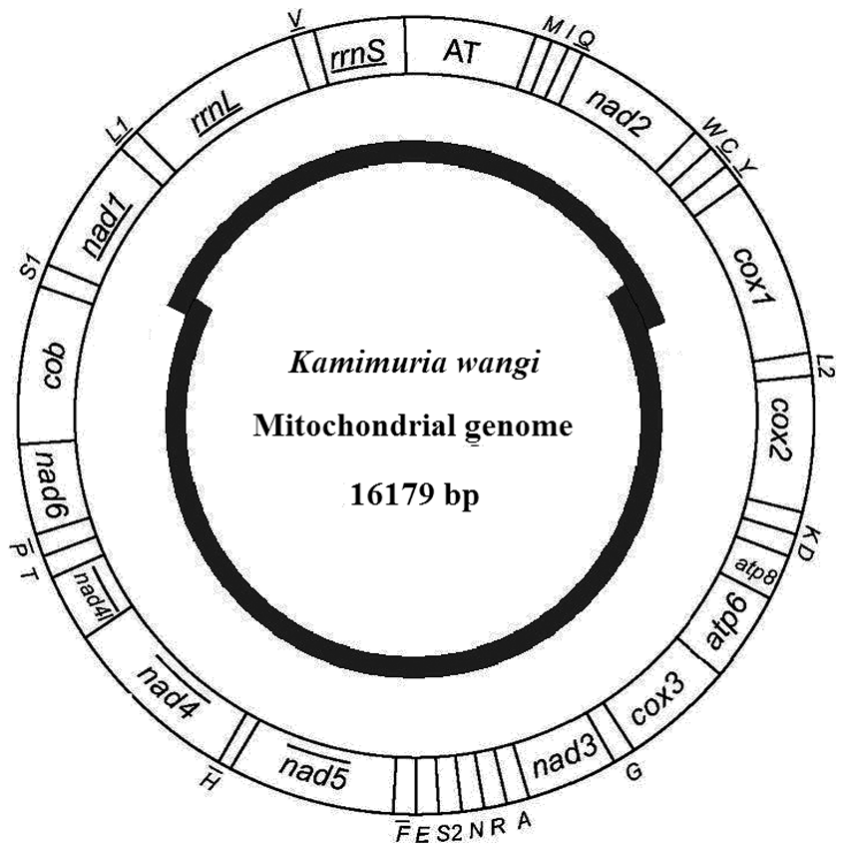
A map of the mitogenome of *Kamimuria wangi*. Transfer RNA genes are designated by single-letter amino acid codes. CR represents the A+T-rich region. The gene name without underline indicates the direction of transcription from left to right, and with underline indicates right to left.

The full-length mtDNA of *K. wangi* is a circular molecule of 16,179 nucleotides, just slightly longer than that of *P. princeps* (16004 bp). The *K. wangi* mtDNA contains 37 genes (13 PCGs, 22 tRNA genes, two rRNA genes) and an AT-rich control region. The number and arrangement of these genes is consistent with insect mitochondrial DNA [5], [15] and identical to the *P. princeps* mitogenome (Figure 1). In 12 locations of the mtDNA, genes overlapped by 1–47 bp (excluding the A+T-rich region; see Table 1). The mtDNA of *K. wangi* also contained intergenic regions (1–28 bp in length) in 13 locations.

**Table 1:** Annotation of the mitochondrial genome of *K. wangi.*

Feature	Region^a^			Length	Codon		IGN^b^
	From	To			Start	Stop	
tRNA^Ile(I)^	1		68	68			
tRNA^Gln(Q)^	(69		137)	69			0
tRNA^Met(M)^	139		207	69			1
ND2	208		1245	1038	ATG	TAA	0
tRNA^Trp(W)^	1245		1311	67			−1
tRNA^Cys(C)^	(1304		1372)	69			−8
tRNA^Tyr(Y)^	(1372		1437)	66			−1
COI	1397		2983	1587	ATA	TAA	−41
tRNA^Leu(UUR)^	2939		3004	66			−45
COII	3010		3705	686	ATG	TAA	56
tRNA^Lys(K)^	3706		3776	71			0
tRNA^Asp(D)^	3778		3846	69			1
ATP8	3856		4005	150	ATA	TAA	9
ATP6	3999		4676	678	ATG	TAA	−7
COIII	4676		5464	789	ATG	TAA	−1
tRNA^Gly(G)^	5467		5533	67			2
ND3	5534		5887	354	ATT	TAA	0
tRNA^Ala(A)^	5889		5953	65			1
tRNA^Arg(R)^	5954		6017	64			0
tRNA^Asn(N)^	6018		6084	67			0
tRNA^Ser(AGN)^	6085		6151	67			0
tRNA^Glu(E)^	6152		6217	66			0
tRNA^Phe(F)^	(6224		6289)	66			6
ND5	(6273		8021)	1749	ATG	TAA	−17
tRNA^His(H)^	(8022		8089)	68			0
ND4	(8091		9455)	1365	ATG	TAG	1
ND4L	(9449		9745)	297	ATG	TAA	−7
tRNA^Thr(T)^	9748		9813	66			2
tRNA^Pro(P)^	(9814		9880)	67			0
ND6	9882		10397	516	ATG	TAA	1
CYTB	10397		11536	1140	ATG	TAG	−1
tRNA^Ser(UCN)^	11535		11604	70			−2
ND1	(11558		12541)	984	ATA	TAA	−47
tRNA^Leu(CUN)^	(12570		12636)	67			28
lr RNA(16s)	(12638		13982)	1345			1
tRNA^Val(V)^	(13991		14061)	71			8
sr RNA(12s)	(14062		14928)	867			0
D-loop	14929		16179	1251			0

a Positions with parentheses indicate the genes encoded by N strand.

b IGN: Intergenic nucleotide; minus (-) indicates overlap between genes.

Thirteen PCGs in *K. wangi* utilize the conventional translational start and stop codons for invertebrate mtDNA. For example, nine PCGs (ND2, COII, ATP6, COIII, ND5, ND4, ND4L, ND6 and CytB) contained ATG codons. Three PCGs (COI, ATP8 and ND1) initiated with ATA codons, and one PCG (ND3) contained ATT as the start site. However, in *P. princeps*, ND2 and ND5 initiated with GTG, ND6 and ATP8 with ATT, and ND1 gene initiated with TTG. Thirteen PCGs used the typical termination codons TAA and TAG in *K. wangi*. In contrast, only two PCGs encode conventional termination codons in *P. princeps*, e.g. TAA for ND4 and TAG for ND1.

The relative synonymous codon usage (RSCU) values showed a biased use of A and T nucleotides in *K. wangi* (Table 2). The high incidence of anticodons NNA and NNU indicated partiality for AT in the anticodon of PCGs. Ile (8.12%), Leu (11.61%), Ser (11.99%) and Thr (7.03%) were the most frequent amino acids in *K. wangi* mtDNA PCGs, and Ser showed the highest incidence. In *P. princeps* PCGs, Ile (7.51%), Leu (12.23%), Asn (7.48%) and Ser (12.15%) were the most common amino acids, and Leu occurred most frequently.

**Table 2:** Codon usage and RSCU of 13 PCGs in the mtDNA of *K. wangi*.

Codon	n(RSCU)	Codon	n(RSCU)	Codon	n(RSCU)	Codon	n(RSCU)
UUU(F)	163(1.33)	UCU(S)	67(1.76)	UAU(Y)	99(1.38)	UGU(C)	10(0.8)
UUC(F)	82(0.67)	UCC(S)	51(1.34)	UAC(Y)	44(0.62)	UGC(C)	15(1.2)
UUA(L)	193(2.31)	UCA(S)	77(2.02)	UAA(*)	98(1.56)	UGA(W)	74(1.68)
UUG(L)	48(0.57)	UCG(S)	13(0.34)	UAG(*)	28(0.44)	UGG(W)	14(0.32)
CUU(L)	99(1.18)	CCU(P)	85(1.5)	CAU(H)	67(1.28)	CGU(R)	14(1.06)
CUC(L)	43(0.51)	CCC(P)	68(1.2)	CAC(H)	38(0.72)	CGC(R)	9(0.68)
CUA(L)	94(1.12)	CCA(P)	60(1.06)	CAA(Q)	89(1.59)	CGA(R)	24(1.81)
CUG(L)	25(0.3)	CCG(P)	14(0.25)	CAG(Q)	23(0.41)	CGG(R)	6(0.45)
AUU(I)	200(1.49)	ACU(T)	89(1.51)	AAU(N)	202(1.47)	AGU(S)	28(0.73)
AUC(I)	68(0.51)	ACC(T)	49(0.83)	AAC(N)	72(0.53)	AGC(S)	19(0.5)
AUA(M)	135(1.56)	ACA(T)	87(1.48)	AAA(K)	210(1.65)	AGA(S)	29(0.76)
AUG(M)	38(0.44)	ACG(T)	10(0.17)	AAG(K)	44(0.35)	AGG(S)	21(0.55)
GUU(V)	65(1.68)	GCU(A)	48(1.28)	GAU(D)	54(1.57)	GGU(G)	29(0.79)
GUC(V)	22(0.57)	GCC(A)	45(1.2)	GAC(D)	15(0.43)	GGC(G)	5(0.14)
GUA(V)	54(1.39)	GCA(A)	50(1.33)	GAA(E)	67(1.58)	GGA(G)	81(2.2)
GUG(V)	14(0.36)	GCG(A)	7(0.19)	GAG(E)	18(0.42)	GGG(G)	32(0.87)

n: the total number of codons.

RSCU: relative synonymous codon usage.

The overall AT content in *K. wangi* mtDNA was 69.6%, which was slightly lower than that of *P. princeps* (70.6%). The average AT content across all PCGs in *K. wangi* was 68.0%, which also lower than that of *P. princeps* (70.5%). The nucleotide composition of the insect mitogenome was biased toward A and T nucleotides. The AT content of the third codon (73.3%) was much higher than the first (63.5%) and second codon positions (67.1%); this suggested that both higher mutation rates and the increased AT occurrence are related and dependent on relaxed selection at the third codon position. However, the A+T% at the second codon (65.7%) was lower than the first (71.0%) and third codon positions (74.9%) in *P. princeps*. Furthermore, we present the detailed comparison in the nucleotide composition, AT-skew and GC-skew between the two stonefly species, *K. wangi* and *P. princeps* (Table 3).

**Table 3:** Comparison of nucleotide composition between *K. wangi* and *P. princeps* mtDNA.

Region	Nucleotides proportion (%)												AT-skew		GC-skew	
	A		T		G		C		A+T		G+C					
	Kw	Pp	Kw	Pp	Kw	Pp	Kw	Pp	Kw	Pp	Kw	Pp	Kw	Pp	Kw	Pp
Whole mtDNA	35.6	36.6	34.0	34.0	11.5	11.5	18.9	17.9	69.6	70.6	30.4	29.4	0.02	0.04	−0.24	−0.22
Protein coding genes	34.7	36.2	33.3	34.3	12.2	11.6	19.8	17.9	68.0	70.5	32.0	29.5	0.02	0.03	−0.23	−0.21
1st codon position	34.8	37.0	28.7	34.0	16.2	13.0	20.3	16.2	63.5	71.0	36.5	29.2	0.10	0.04	−0.11	−0.11
2nd codon position	31.2	32.7	35.9	33.0	11.0	14.1	21.9	20.1	67.1	65.7	32.9	34.2	−0.07	−0.01	−0.33	−0.18
3rd codon position	38.0	38.9	35.3	36.0	9.5	7.8	17.2	17.4	73.3	74.9	26.7	25.2	0.04	0.04	−0.29	−0.38
tRNA genes	36.8	35.8	35.6	34.3	12.1	13.2	15.4	16.8	72.4	70.1	27.5	30.0	0.02	0.02	−0.12	−0.12
lrRNA	38.5	39.8	34.6	34.2	8.8	8.1	18.1	17.9	73.1	74.0	26.9	26.0	0.05	0.08	−0.35	−0.37
srRNA	37.0	37.3	30.6	31.3	11.0	11.6	21.5	19.8	67.6	68.6	32.5	31.4	0.09	0.09	−0.32	−0.26
A+T-rich region	38.8	43.9	39.4	37.4	7.6	6.4	14.2	12.3	78.2	81.3	21.8	18.7	−0.01	0.08	−0.30	−0.32

Note: Kw indicates *K. wangi* and Pp indicates *P. princeps*.

**Figure 2:**
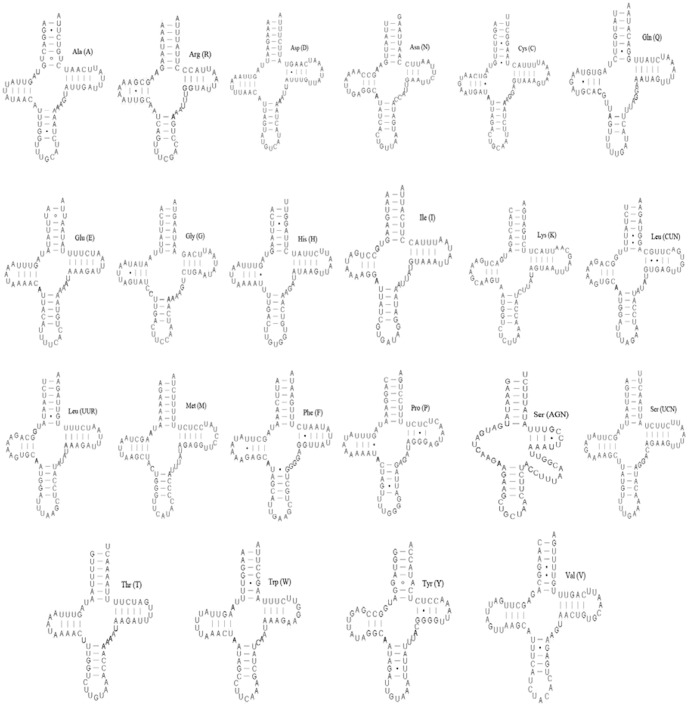
Predicted secondary structure for the 22 typical tRNA genes of *K. wangi*. Dashes (–) indicate Watson-Crick bonds, and dots (•) indicate mistaken bonds.

The mitogenome of *K. wangi* had 22 traditional tRNA genes interspersed with rRNAs or PCGs. The tRNA genes were encoded by a total of 1484 nucleotides; tRNAs ranged from 64 to 71 bp and the A+T content was 72.4%. Fourteen tRNAs were encoded by the H-strand and the remaining eight were encoded by the L-strand. All tRNA genes had the typical cloverleaf secondary structure except for the tRNA^Ser(AGN)^ gene; in this molecule, the stable stem-loop structure of the dihydrouridine (DHU) arm was missing (Figure 2), a feature that has been observed in other metazoan mitogenomes [16], [17].

**Figure 3:**
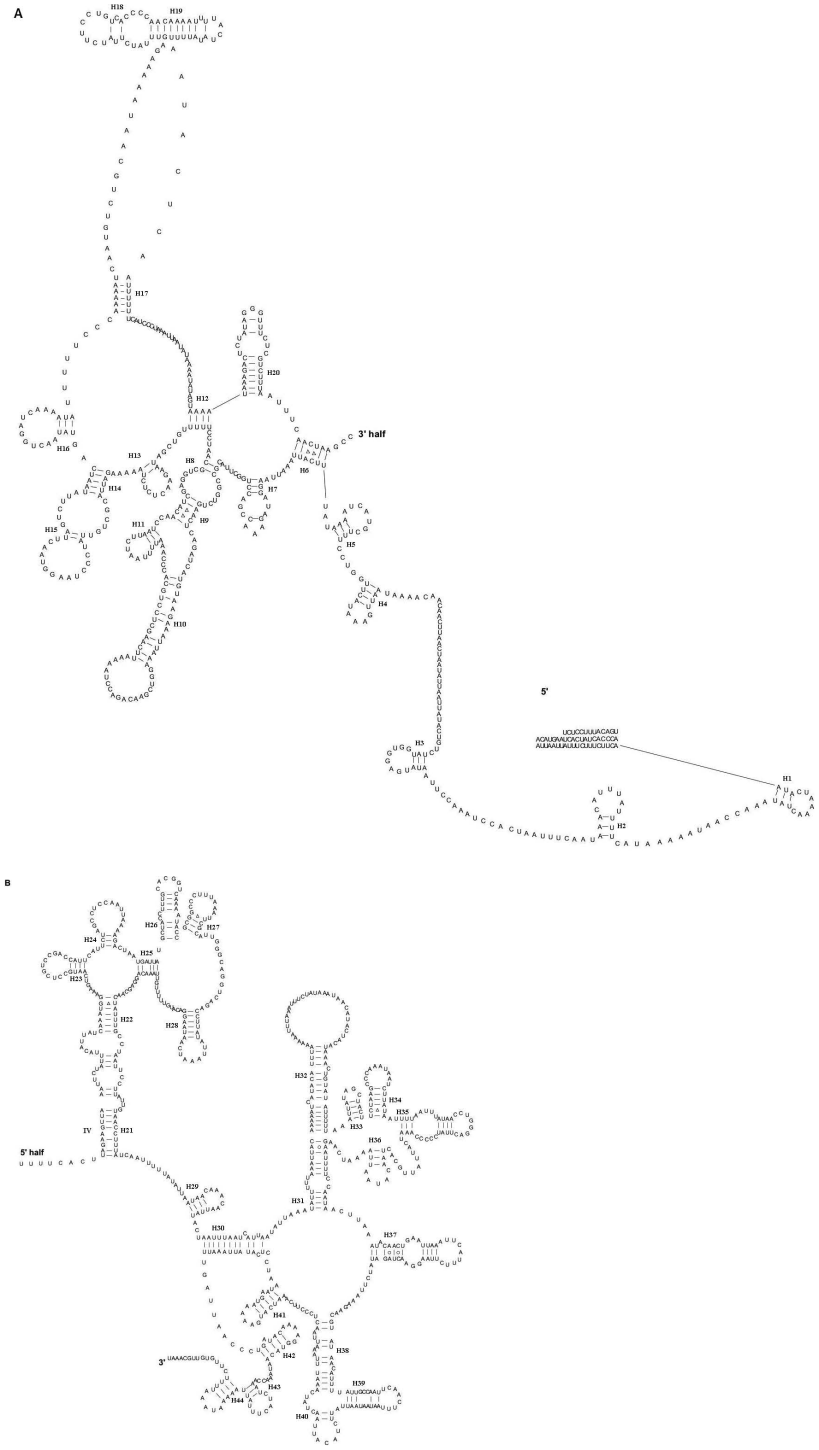
Predicted lrRNA secondary structure in *K. wangi* mitochondrial genome. A indicates 5′ half of lrRNA; B indicates 3′ half of lrRNA. Base-pairings are indicated as follows: the box-, circle- and line-drawings in the figure indicates the tertiary structure bonds. Roman numerals denote the domain structure. Dashes (–) indicate inferred Watson-Crick bonds, and dots (•) indicate mistaken bonds.

**Figure 4:**
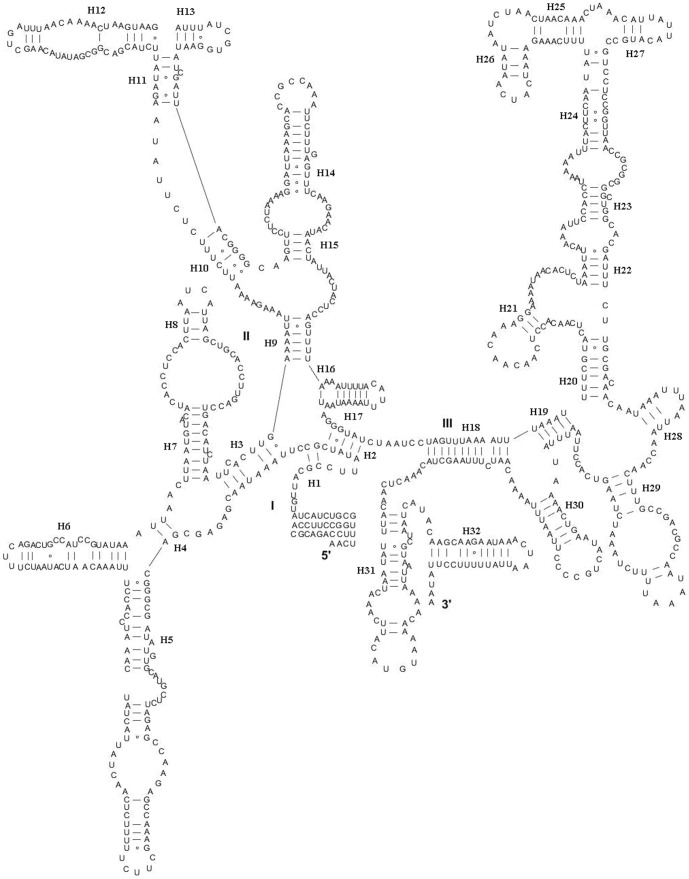
Predicted srRNA secondary structure in *K. wangi* mitochondrial genome. The annotation is the same as in Fig. 3.

There were two rRNAs in *K. wangi* with a combined length of 2221 bp and an A+T content of 71.0%. The lrRNA gene (*rrnL*) had a length of 1345 bp, an A+T content of 73.1%, and was positioned between tRNA^Leu(CUN)^ and tRNA^Val^. The srRNA gene (*rrnS*) was 867 bp, had an A+T content of 67.6%, and was located between the tRNA^Val^ and A+T-rich region. Gene sizes and map positions were consistent with those observed in the mitogenomes of other insects. Furthermore, both the AT- and GC-skews were slightly positive in the tRNA and rRNA genes, which correlates with the results obtained for *P. princeps*. The secondary structures of lrRNA and srRNA were consistent with the models proposed for these genes in other insects [18]–[20]. In *K. wangi*, the lrRNA gene contained six domains (labeled I, II, III, IV, V and VI) with 44 helices (Figure 3). The srRNA gene contained three domains (labeled I, II, III) and 32 helices (Figure 4).

**Figure 5:**
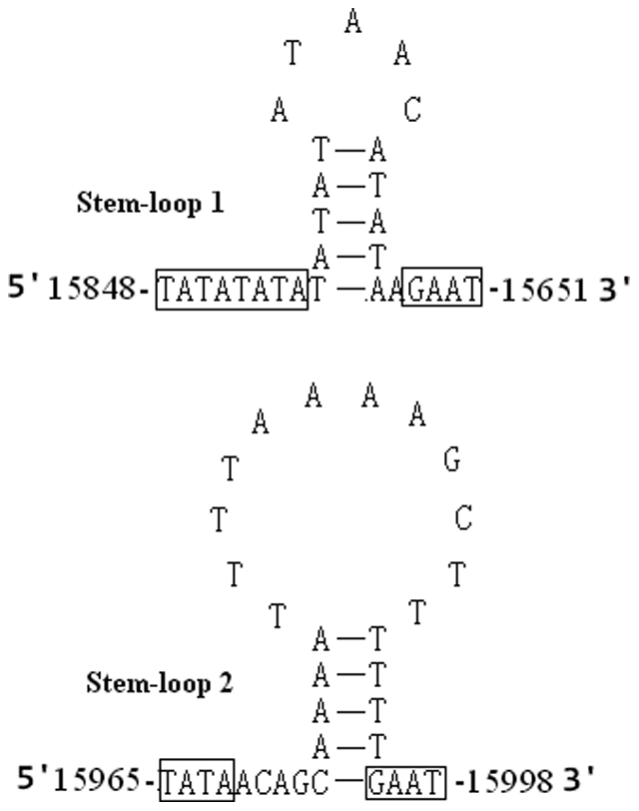
The potential stem-loop structure found in the *K. wangi* A+T-rich region. The motifs (“(TA)_n_” and “GAAT”) in the flanking region of the stem-loop structures are indicated by boxing [43].

Previously, the A+T-rich region was reported to contain elements essential to the initiation of replication and transcription [21]. The A+T-rich region of the *K. wangi* mitogenome was 1251 bp and mapped between the srRNA and tRNA^Ile^-tRNA^Gln^-tRNA^Met^ gene cluster (Figure 1). The A+T content of the AT-rich region was 78.2%, slightly lower than the corresponding region in *P. princeps* (81.3%). Additionally, there was a 10-bp poly-T stretch observed in the A+T-rich region of *K. wangi*. Two regions located downstream of the poly-T stretches were identified and designated stem-loop 1 (positions 15848–15651) and stem-loop 2 (positions 15965–15998) based on their potential ability to fold into stem-loop structures (Figure 5).

### Phylogeny of basal Neoptera

In this study, the amino acid sequences of the 13 PCGs were concatenated to construct phylogenetic relationships, which may result in a more complete analysis than analyzing each sequence separately. We incorporated species from Orthoptera, Ephemeroptera, Blattodea, Mantodea, Mantophasmatodea, Phasmatodea, Lepidoptera, Embioptera, Hemiptera, and Hymenoptera in the phylogenetic analysis (Table 4). Zoraptera and Dermaptera were not included in the analyses because mtDNA data are incomplete for these groups. Odonata was included as an outgroup.

**Table 4:** List of taxa and mtDNAs analyzed in this study.

Order	Species	Length (bp)	Acc. number
Hymenoptera	*Orussus occidentalis*	15847	FJ478174
Lepidoptera	*Ochrogaster lunifer*	15593	AM946601
Mantophasmatodea	*Sclerophasma paresisense*	15500	DQ241798
Orthotera	*Schistocerca gregaria*	15625	GQ491031
Phasmatodea	*Ramulus hainanense*	15590	FJ156750
Blattodea	*Blattella germanica*	15025	EU854321
Mantodea	*Tamolanica tamolana*	16055	DQ241797
Hemiptera	*Schizaphis graminum*	15721	AY531391
Plecoptera	*Pteronarcys princeps*	16004	AY687866
	*Kamimuria wangi*	16179	KC894944
Ephemeroptera	*Siphlonurus immanis*	15529	FJ606783
Odonata	*Euphaea formosa*	15700	HM126547
Embioptera	*Aposthonia japonica*	18305	AB639034

**Figure 6:**
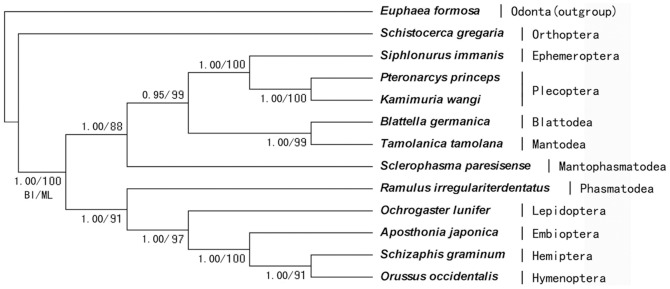
Phylogenetic trees based on mitogenome. Phylogenitic tree inferred from amino acid sequences of 13 PCGs of the mitogenome by using Bayesian inference (BI), Neighbor Joining (NJ) and Maximum Likelihood (ML). Numbers at each node indicate bootstrap support; percentages of NJ probabilities (first value) and ML bootstrap support values (second value), respectively. *Euphaea formosa* was used as outgroup.

Analysis using BI and ML resulted in two trees with the same topology except for some variation in node confidence values (Figure 6). Numbers at each node indicate bootstrap support, percentages of Bayesian posterior probabilities (first value) and ML bootstrap support values (second value).

The results support the Embioptera was a sister group of a clade containing Hemiptera and Hymenoptera. *S. immanis* (Ephemeroptera) grouped with the mitogenomes of *P. princeps* and *K. wangi* (Plecoptera) in Polyneoptera; this grouping differed from conclusions based on morphological classifications and some molecular studies, but was consistent with the findings of Zhang et al. [8] and Lin et al. [9].

Plecoptera are traditionally associated with the lower Neoptera and often placed within Polyneoptera. There has been very little consensus regarding the placement of Plecoptera within this group, which is comprised of ten other orders: Blattodea, Dermaptera, Embiidina, Grylloblattodea, Isoptera, Mantodea, Mantophasmatodea, Orthoptera, Phasmatodea and Zoraptera [22]–[24]. Boudreaux placed Plecoptera within Polyneoptera as the sister taxon to Embiidina [11]. Henning recognized all polyneopterous orders except Plecoptera as a monophylectic group and assigned it to the Paurometabola [12]. However, he was unable to assign Plecoptera and left it as a lineage disconnected from the overall topology. Kukalova-Peck depicted Polyneoptera as paraphyletic and placed Plecoptera as the sister taxon to the “Orthopteroid orders” [25]. However, Kristensen leaves Polyneoptera (including Plecoptera) as a largely unresolved polytomy at the base of Neoptera [22]. Molecular studies generally place Plecoptera as a sister to Dermaptera based on the gene sequences encoding the small subunit of nuclear ribosomal DNA [26]. Wheeler et al. used both molecular and morphological data to support a monophyletic Polyneoptera and placed Plecoptera as sister taxon to Embiidina [24]. However, Terry & Whiting [27] used sequences derived from 18S rDNA, 28S rDNA and histone 3 to assign Plecoptera as a sister lineage to Dermaptera and Zoraptera. The evolution of hemocyanin subunits follows the widely-accepted phylogeny of the Hexapoda and provides strong evidence for the monophyly of the Polyneoptera (Plecoptera, Dermaptera, Orthoptera, Phasmatodea, Mantodea, Isoptera, Blattaria), which subsequently places Plecoptera as a sister taxon to Embiidina and Zoraptera [28]. The resulting trees strongly support monophyly of Neoptera and Polyneoptera and place Plecoptera as the sister taxon to Dermaptera based on three nuclear protein-coding genes [29].

However, our phylogenetic analyses show an unexpected monophyletic Plecoptera and Ephemeroptera clade sister in Polyneoptera. This result is so interesting, because the two taxa share aquatic life history of naiads and a few studies have used stoneflies as models for understanding the origin of flight in early winged insects [30].

The phylogenetic clustering of the Plecoptera with Ephemeroptera was unexpected, may be a result of taxon sampling at the base of the pterygotes. Broad and sufficient taxon sampling is an important factor in phylogenetic analyses of insect mitochondrial genomes. Accordingly, it is necessary to increase the number of representative species within the target taxa, excluding the highly divergent genomes with variable genome rearrangements, elevated substitution rates, or base compositional bias, which likely cause long branch attraction. Our phylogenetic trees cannot illustrate phylogenetic system of Neopteran insects completely and clearly, because the missing mtDNA sequences from Dermaptera and Zoraptera.

## Materials and Methods

### Ethics statement of Animals

There were no special permits for the insect collection for this study in Henan Province, China. The insect samples used in this experiment were collected from a clean creek flowing from the mountain. The field studies did not involve endangered or protected species. The species in the genus of *Kamimuria* are common small aquatic insects and are not included in the “List of Protected Animals in China”.

### Sample preparation and DNA extraction

Specimens of *K. wangi* were collected at Luoyang in Henan Province (33.70° N, 111.84°E) in July 2009, China. All specimens were identified and then preserved in 100% ethanol and stored at −70°C until DNA extraction was performed. Genomic DNA was extracted from individual audult samples using the Axygen DNA kit (Axygen Biotechnology Hangzhou, China) and used as a template for LA-PCR.

### PCR amplification and sequencing

Two pairs of LA-PCR primers were used to amplify two overlapping portions of mtDNA in *K. wangi*. The LA-PCR primer sequences were as follows: A1: 5′-CGAGCWTACTTTACTTCAGCMACWATAATTA-3′and A2: 5′-GCAAATAR RAATRATCATTCATTCTGGTTGGAT-3′; B1: 5′-TACACCAAACAGGATCAAAT AAYCCMWTAGG-3′and B2: 5′-GCTCCAACATRTTTCTRCATTGACCAAATA-3′. The LA-PCR amplifications were performed with LA *Taq* DNA polymerase (Takara, Japan) in an ABI thermal cycler using the following program: initial denaturation at 93°C for 2 min, followed by 40 cycles at 92°C for 10 s, annealing at 54°C for 30 s, elongation at 68°C for 8 min (20 cycles), and increased 20 s per cycle in the last 20 cycles. The final elongation step was continued at 68°C for 7 min. LA-PCR products were purified with an Axygen DNA Gel Extraction Kit (Axygen Biotechnology Hangzhou, China) after separation by electrophoresis in 1.0% agarose gels.

Additional PCR primers (shown in Table S1) were designed by comparing mtDNA sequences from 30 Neopteran insect species (including Plecoptera) with universal primers of insect mtDNA [5]. These amplifications were performed using templates from LA-PCR as follows: primary denaturation at 94°C for 2 min, 35 cycles at 94°C for 30 s, annealing at 40–55°C for 30 s, extension at 72°C for 90 s, and final extension at 72°C for 7 min. All PCR fragments from *K. wangi* were sequenced after separation and purification.

Purified PCR products were ligated into pGEMT Easy Vector (Promega). Recombinant clones were sequenced in both direction using the BigDye Terminator Sequencing Kit (Applied BioSystems) and the ABI 3730XL Genetic Analyzer (PE Applied Biosystems, San Francisco, CA, USA) with two vector-specific primers and internal primers for primer walking. The mitogenome of *K. wangi* was amplified from overlapping PCR fragments. In order to guarantee the quality of sequences, each PCR fragment contained five copies and the sequences were checked by the Chromas. All well sequenced fragements were assembled using ContigExpress and DNAMAN and then were blasted in NCBI to identify the purpose fragements.

### Genome annotation and secondary structure prediction

We used CodonCode Aligner for sequence assembly and annotation. Transfer RNA genes were initially identified using the tRNAscan-SE software available online at http://lowelab.ucsc.edu/tRNAscan-SE/ [31]. PCGs and rRNA genes were identified by comparison with the *P. princeps* mitochondrial genome. The software of XRNA 1.2.0.b (http://rna.ucsc.edu/rnacenter/xrna/xrna.html) was used to draw the secondary structure of tRNA genes. The secondary structures of *rrnL* and *rrnS* genes were inferred based on models developed for other insect species [32]–[35]. To infer the secondary structures of tRNA and rRNA molecules, we used a commonly accepted comparative approach to correct for unusual pairings with RNA-editing mechanisms that are well known in arthropod mitogenomes [36], [37]. AT-skew and GC-skew were calculated to determine the AT and GC biases of PCGs in *K. wangi* and *P. princeps*, using the following formulas: AT-skew  =  [A−T]/[A+T]; and GC-skew  =  [G−C]/[G+C] [38]. Translated amino acid sequences of the PCGs were aligned using ClustalW [39] in MEGA 5 [40] after eliminating initiation codons. The AT content and codon usage were calculated using MEGA 5.

### Phylogenetic analysis

To examine the phylogenetic position of stonefly, 12 additional Neopteran mitogenomes were downloaded from GenBank and compared using the mitogenome of Odonata (*Euphaea formosa*) as the outgroup (Table 4). The amino acid sequences of the 13 PCGs were aligned with ClustalX using default settings and concatenation [41]. The best-fitting model by Modeltest using likelihood ratio tests, was then used to perform Bayesian inferences (BI) and maxi-mum likelihood (ML) analysis using the program MrBayes 3.1.2 (http://morphbank.ebc.uu.SE/mrbayes/) and MEGA version 5.0 software with maximum likelihood (ML) methods [42]. DNA alignments were inferred from the amino acid alignment of 13 PCGs using default settings in ClustalX and MEGA version 5.0, which could alternate between DNA and amino acid sequences within alignments. Alignments of individual genes were then concatenated without the stop codon. The best fit model for nucleotide alignments was determined by Modeltest 3.7. According to the Akaike information criterion, the GTR + I + G paradigm was the most ideal model for analysis using nucleotide alignments. The BI analyses were conducted under the following conditions: 1,000,000 generations, four chains (one cold chain and three hot chains) and a burn-in step for the first 10,000 generations. The confidence values of the BI tree were expressed as the Bayesian posterior probabilities in percentages. The ML methods bootstrap analysis was done with 1000 replications, and values were calculated using the 50% majority rule.

## Supporting Information

Table S1 **Additional primers used in this study.**
(DOC)
