# Supplementary material for: Mitochondrial Genome of the Stonefly Kamimuria wangi (Plecoptera: Perlidae) and Phylogenetic Position of Plecoptera Based on Mitogenomes
Source: PLoS One. 2014 Jan 23;9(1):e86328. doi: 10.1371/journal.pone.0086328 (PMC3900512; doi:10.1371/journal.pone.0086328)
Supplement: Table S1 — Additional primers used in this study. (DOC) [file pone.0086328.s001.doc]

**Table S1 Additional primers used in this study**

| **Fragment codes** | **Primers** | **Sequences** |
| --- | --- | --- |
| A01 | U2466 | CGAGCRTACTTTACTTCACGMACWAT  AATTA |
|  | D3057 | TAWCTATGGTCTTCAGGKGG |
| A02 | U2830 | TAACATTCTTYCCAACTCAYTT |
|  | D3647 | TAATTGTTCTATTAAAGGGGA |
| A03 | U3552 | ATTTAACTCGYAWAWATAAAG |
|  | D4184 | GCTCCAARACTTTCTRCATTGACCAAARAA |
| A04 | U4124 | GATGCCCACAWAGGWCGAYT |
|  | D5022 | ATTCAWRCCTATATTATAAA |
| A05 | U4674 | AAYTGAAAAATAGTACAAAYYTWTT |
|  | D5553 | AYATCWCTGCATCATTGRATTAT |
| A06 | U5433 | AGACCTCCAGTMTTAACWGGAGC |
|  | D6158 | CTACCTCATAATRACATTGA |
| A07 | U6103 | GCAGCWGCAGTWTAYTGACA |
|  | D6736 | AGGTCGAAGTCAARTGCAA |
| A08 | U6598 | GAATGAAAGAACYGAGCWYT |
|  | D7077 | AGYCARTAAYTGAAGATGTT |
| A09 | U6993 | AAAACATTACATTTTCAYTGT |
|  | D7771 | TTTTAYTCWAATTAGGTAAT |
| A10 | U7699 | GTTAAGACCMTAGAAAAAAAA |
|  | D8444 | ARTGCAKGGTTATTAACTGC |
| A 11 | U8368 | ATAATTTYCAACWCCAAAAT |
|  | D9226 | AATAKCGATGCWCCTCCTTC |
| A 12 | U9009 | AWATTTAAAGGCYMAAAATG |
|  | D9720 | TTTTTAGTWARAATRCCAATATT |
| A 13 | U9678 | GGTYCGRCAACATGAGCYTT |
|  | D9921 | TTAATTTTGYKGGRAGGKTA |
| A 14 | U9876 | GTATAAAAAAKTAMATAAAC |
|  | D10641 | GRTTTACACCAGARRTGTTTT |
| A 15 | U10393 | ATCCCCAAAATAAYRATTTT |
|  | D10946 | AATAAAACTAMKGTACCWCC |
| A 16 | U10726 | ATCCCCAAATAAYYRAATTTT |
|  | D11518 | CCATWATTTAGATCTCGRCA |
| A 17 | U11456 | TTATTYTCGATWATACATTA |
|  | D12121 | GCAAATARRAARTATCATTCATTCTGGTTGGAT |
| B18 | U12068 | TAGGWGAWCYYGATAAYTTT |
|  | D12854 | TCTGGGTTRTAATTKGARTAT |
| B19 | U12668 | TTATCAGCWATAAAWCGAGG |
|  | D13385 | ATTCGWTGARAATCCTAATAA |
| B20 | U13342 | ATTGCATAWCRCAAAGGYTG |
|  | D14031 | GGACGAGACAGACCTATAGA |
| B21 | U13842 | ACTCTCGTATCCCTAAGGTA |
|  | D14358 | GTAATAATATAGAAATTAGT |
| B22 | U14130 | AACCATTACATCYAGCCTYCAATT |
|  | D14779 | CTTKGATGACAGGGTTATWAA |
| B23 | U14621 | AAATTYWTTTARTTCRAAAC |
|  | D15177 | AAAYAYGRTCACATCGCCCGT |
| B24 | U15097 | CTACGTATTTACGACTTATCT |
|  | D15632 | GGTTGTTGAAKCTAGTKAATTT |
| B25 | U15606 | GGGTATMATACCTATAGTTTA |
|  | D16368 | GTTGATGTGGTAAATTTATTGTC |
| B26 | U16462 | GCCCCAYWTRTTAAATAAAT |
|  | D116 | GCACRWTGARTTTTGATACT |
| B27 | U70 | ACCTTCATRWAGTYMAATAG |
|  | D1037 | ATTCAYAATAWRGRAAATCC |
| B28 | U1004 | TACTATCMCGGWTTGGAYTA |
|  | D1626 | GATGTTCCTCAWATTCCTGMTCA |
| B29 | U1580 | TCTCACACYAGAYAAGGAYA |
|  | D2486 | ACAGCAATAATRTATWGTKGC |
